# Genomic Instability Is Associated with Natural Life Span Variation in *Saccharomyces cerevisiae*

**DOI:** 10.1371/journal.pone.0002670

**Published:** 2008-07-16

**Authors:** Hong Qin, Meng Lu, David S. Goldfarb

**Affiliations:** 1 Center for Aging and Development Biology, Department of Biology, University of Rochester, Rochester, New York, United States of America; 2 Department of Biology, University of Rochester, Rochester, New York, United States of America; University of Minnesota, United States of America

## Abstract

Increasing genomic instability is associated with aging in eukaryotes, but the connection between genomic instability and natural variation in life span is unknown. We have quantified chronological life span and loss-of-heterozygosity (LOH) in 11 natural isolates of *Saccharomyces cerevisiae*. We show that genomic instability increases and mitotic asymmetry breaks down during chronological aging. The age-dependent increase of genomic instability generally lags behind the drop of viability and this delay accounts for ∼50% of the observed natural variation of replicative life span in these yeast isolates. We conclude that the abilities of yeast strains to tolerate genomic instability co-vary with their replicative life spans. To the best of our knowledge, this is the first quantitative evidence that demonstrates a link between genomic instability and natural variation in life span.

## Introduction

Accumulation of oxidative damage and loss of genomic integrity likely contribute to the finite life spans of organisms from yeast to humans. In mammals, increasing genomic instability is also associated with age-related diseases such as cancer [1], [2]. In the budding yeast *Saccharomyces cerevisiae*, aging can be studied using both replicative and chronological measures of life span [3], [4]. Replicative life span (RLS) is the number of daughter cells that single mother cells can produce before they senesce and die [5], [6]. Daughter cells of aging mother cells are generally born rejuvenated due, in part, to the partition of damaged proteins to mother cells [7]. This mitotic asymmetry breaks down in very old mother cells [8]. Hence, daughter cells of older mother cells are relatively shorter-lived than those of younger mother cells [9] and their genomes become increasingly unstable, as measured by loss-of-heterozygosity (LOH) [10], [11]. Mutations that disrupt mitotic asymmetry are known to shorten the replicative life spans of both mother and daughter cells [7], [12].

Chronological life span (CLS) is typically measured by the colony forming capacity of non-dividing cells as a function of time [3], [13], [14]. Age-dependent decreases in genomic integrity, accumulation of reactive oxygen species (ROS), and oxidative damage to specific proteins are common features of both replicative and chronological models of aging [8], [11], [15], [16].

Genetic influence on life span plasticity is of great interest, as highlighted by studies in human populations [17]–[19]. Both RLS and CLS of natural yeast populations vary considerably [20]. Significant differences in life span also exist between laboratory strains and natural isolates of yeast [20]. Hence, investigating the mechanism underlying natural life span variation of yeast can help us better understand the natural causes of aging and life span plasticity. Although genomic instability is linked with the aging process by ample evidences [1], [2], [11], its link with natural variation in life span has not been demonstrated. We hypothesize that the capacity of different yeast strains to maintain genomic integrity and mitotic asymmetry during aging may be an underlying cause of, or at least correlated with variation in natural life span. In the present study, we have tested this hypothesis through quantitative studies of genomic instability and mitotic asymmetry during yeast chronological aging using a collection of yeast natural isolates.

## Results

### Overview of the experiment

**Figure 1:**
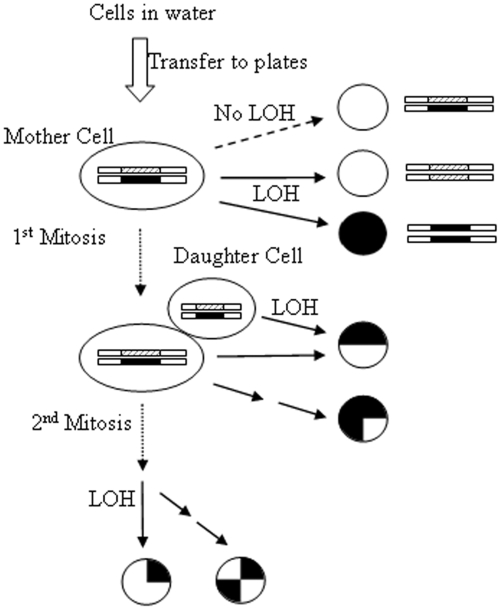
Schematic diagram of LOH assay. One copy of the wild-type allele at the *MET15* locus was knocked out by a Kanamycin-resistance marker (The white box with gray lines is the *MET15* ^+^ allele, and the dark box is the *met15* ^−^ allele). Each solid arrow represents a single LOH event. Colonies formed by *MET15* ^+/+^ and *MET15* ^+/−^ are light-colored on Pb^2+^-containing plates. Colonies formed by met15^−/−^ are black. LOH occurred after the first mitosis can lead to half- and quarter-black colonies. Full-blacks are due to LOH in mother cells. Half-blacks may be due to LOH in either mother or daughter cells between the 1^st^ and 2^nd^ mitoses. The term “mother” and “daughter” cells are used here with respect to the timing of LOH. Mitosis occurs after cells have been transferred to plates. Hence, “mother” cells refer to all the cells maintained in stationary phase in water.

The conversion of heterozygous *MET15* ^+/−^ cells to homozygous *met15* ^−/−^ is a detectable LOH event [21] (Figure 1). On Pb^2+^-containing plates, heterozygous *MET15* ^+/−^ or homozygous *MET15* ^+/+^ cells form light-colored colonies, whereas homozygous *met15* ^−/−^ cells produce black or dark-brown colonies. Therefore, the frequency of dark colonies arising from a population of *MET15* ^+/−^ cells represents half of the LOH events.

To investigate the effect of aging on LOH, CLS assays were performed in water with *MET15* ^+/−^ cells. At different time intervals, aliquots of the cell cultures were spread on Pb^2+^-containings plates after serial dilutions [21] and scored after 2 days of incubation at 30°C for whites, full-blacks, half-blacks, quarter-blacks, or three-quarter-blacks. Full-black colonies arise from cells that underwent an LOH event while suspended in water (Figure 1). Sectioned black colonies indicate that LOH occurred after the cells had passed the first cell division on Pb^2+^-containing plates. The full-black, half-black, and quarter-black colonies generally occurred in 5∼10 fold decreasing frequencies. Notice that the terms “mother” and “daughter” cells are defined here with respect to the timing of LOH events during mitosis on plates.

Loss of viability during chronological aging is modeled using a logistical function as previously described [20], [22]:
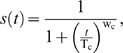
(1)where *t* is time, *s*(*t*) is viability as a function of time, T_c_ is the chronological life span defined at the time when *s*(*t*) is 50%, and w_c_ is a weight parameter. (A list of key variables is summarized in Table 1.)

**Table 1:** Summary of key variables.

*t*	Time measured in days.
*s*, *s*(*t*)	Viability as a function of time (t).
*g*, *g*(*t*)	Genomic integrity as a function of time (t).
T_c_	Chronological life span measured on Pb^2+^-containing plates.
T_g_	The time point that *g*(*t*) reaches the midpoint of its total change.
w_c_	The weight parameter in the logistic model for chronological life span.
w_g_	The weight parameter in the logistic model for genomic integrity.
*m*(*t*)	Mortality rate, i.e., the normalized declining rate of viability s(t).
*r*(*t*)	Rate of genomic instability, i.e., the normalized declining rate of g(t).
T_mmax_	Time point when *m*(*t*) peaks.
T_rmax_	Time point when *r*(*t*) peaks.
T_dmax_	The time point that *b_1/2_*(*t*) reaches its maximum.
*b*, *b*(*t*)	Percentage of full-black colonies as a function of time.
b_max_	Maximum of *b*(t).
b_min_	Minimum of *b*(t).
*b_1/2_*(*t*)	The chance that wild type cells generate half-black colonies.
*L*(*t*)	Ratio of half-blacks over full-blacks over time.
L_0_	L(t) at time zero, a measure of initial mitotic asymmetry.
L_max_	Maximum of L(t).

We introduced a heterozygous *MET*15^+/−^ marker into 11 natural isolates and quantified changes of LOH and CLS among these strains. Each strain was assayed at least 3 times. Among the 3 independent experiments for each strain (except one isolate), one was done using a different clone obtained during strain constructions.

### A model to quantify LOH in chronological aging

**Figure 2:**
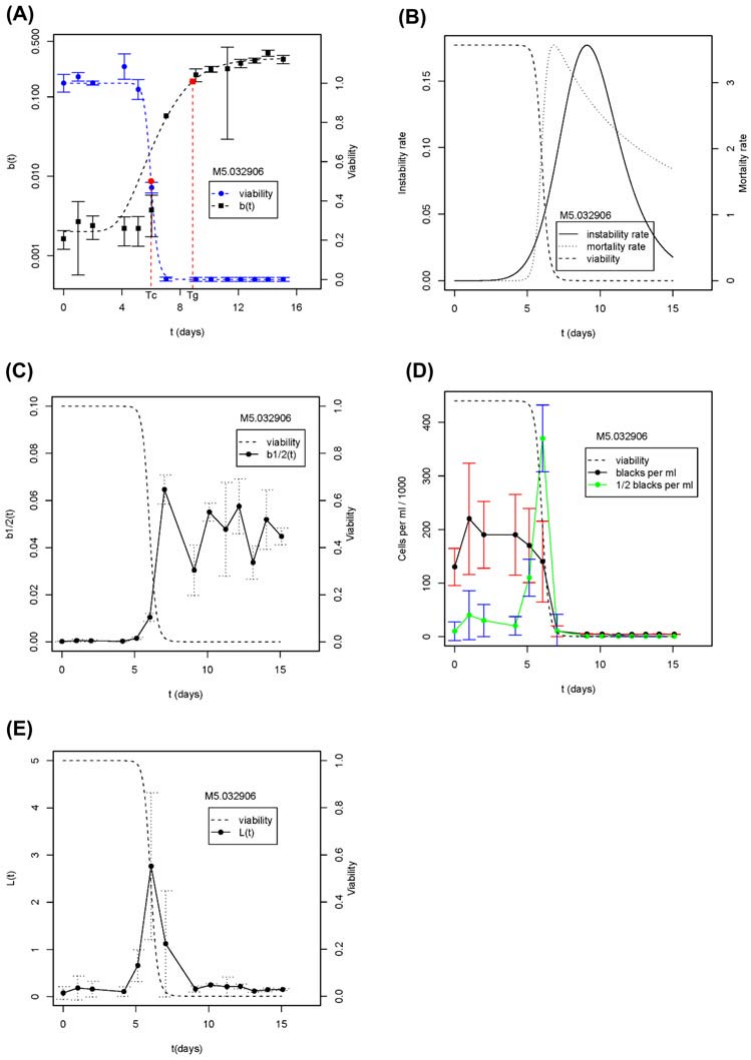
Change of genomic instability during chronological aging. A representative experiment is presented for strain M5. (A) Change of viability (*s*) and full-black colonies (*b*) are in percentage over time and are shown in different scales. The chronological life span is estimated at T_c_. T_g_ indicates the time when *b*(*t*) reached its midpoint. (B) Changes of the mortality rate and the genomic instability rate in chronological aging. (C) Change of half-black colonies in fraction over time. The time that *b*_1/2_(*t*) peaks are calculated using weighted averaged from a 3-point-window around the observed peaks. (D) Change of full- and half-black colonies in frequency over time. Frequencies are calculated for 1 ml of water. (E) Change of *L*(*t*) suggests that mitotic asymmetry changes over time. In all panels, error bars indicate standard deviation calculated from 3 plates.

As shown in Figure 2A, the percentage of black colonies over time is sigmoid-shaped. This sigmoid increase can be approximately modeled using a logistical function, similar to the increase of dead cells during aging. In order to be comparable to our CLS model based on the decrease of viability (Eq 1), we modeled the decrease of genomic integrity as a function of time, assuming both types of LOH outcomes (*MET*15^+/+^ and *met*15^−/−^) occur with the same frequency. This implies:
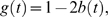
(2)where *g*(*t*) is genomic integrity measured at the *MET15* locus as a function of time and *b*(*t*) is percentage of black colonies as a function of time. Hence, we define genomic integrity as the percentage of cells that have not undergone LOH. This approach yields the following model for *b*(*t*):
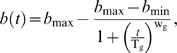
(3)where b_max_ is the maximum of *b*(*t*) that can be reached when *b*(*t*) plateaus at old ages, b_min_ is the initial percentage of full-blacks, and T_g_ is the time when the decrease of genomic integrity reaches 50% of its total extent of changes (Detailed in Methods). T_g_ and T_c_ are conceptually comparable, both representing the midpoints of the changing processes that they describe.

### Age-dependent increase of LOH in mother cells

The decrease of viability and the increase of genomic instability during CLS for 11 natural isolates were quantified using Eq 1 and 3. The estimates of T_c_ and T_g_ for all 11 strains are listed in Table S1. As can be seen from Figure 2A, the percentage of full black colonies, *b*(*t*), increases dramatically during the die-off phase of chronological aging. A temporal lag between the rapid loss of viability and the rapid increase in LOH is described as the difference between T_c_ and T_g_, which for strain M5 is ∼1.9 days. In fact, T_c_ is strongly correlated with T_g_ for all 11 strains (R^2^ = 0.59 and p-value = 0.006, Table 2), indicating the existence of a physiologically relevant, strain-independent relationship between chronological aging and genomic instability. For these 11 strains T_g_ is 1.4±0.5 fold of T_c_ (pair-wise t-test, p-value = 0.001). An analogous shift to significantly higher frequencies of LOH among daughter cells was previously reported during replicative aging [10], [11].

**Table 2:** The R^2^ and p values of key pair-wise correlations on natural life span variation and genomic instability.

	ARLS	CLS	T_c_	T_g_	T_dmax_	T_mmax_	T_rmax_	T_g_/T_c_	T_rmax_/T_mmax_	b_max_
CLS	—									
T_c_	—	—								
T_g_	—	—	0.59; 0.006							
T_dmax_	—	—	0.71; 0.001	0.51; 0.01						
T_mmax_	—	—	—	0.61; 0.004	0.76; 0.0005					
T_rmax_	—	—	0.59; 0.006	—	0.48; 0.02	0.60; 0.005				
T_g_/T_c_	0.56; 0.008	—	—	—	—	—	—			
T_rmax_/T_mmax_	0.52; 0.01	—	—	—	—	—	—	—		
b_max_	0.34; 0.06	—	—	0.33; 0.06	—	—	0.34; 0.06	0.35; 0.05	0.35; 0.06	
L_0_	—	*0.53*; *0.01*	—	—	—	—	—	—	—	—

In each cell, the first value is R^2^ and the second one is p-value. Pair-wise correlations are based on linear regressions. Except L0∼CLS (in italic), all other correlations are positive. Only correlations with appreciable p-values are presented. Some correlations are excluded because they involve self-derived parameters. Partial correlation among ARLS, T_g_/T_c_ and b_max_ are also significant but are omitted for clarity. ARLS and CLS values are both measured using YPD media and are taken from previous publications [20].

Another way to quantify aging is to measure mortality rate over time. Mortality rate, *m*(*t*), can be defined as the normalized declining rate of viability, *s*(*t*),
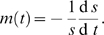
(4)Likewise, we adopted a similar measure to evaluate the normalized declining rate of genomic integrity:
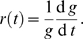
(5)This measure, *r*(*t*), is termed the rate of genomic instability.

As shown in Figure 2B, *r*(*t*) and *m*(*t*) for strain M5 are temporally correlated. To compare the changes of the two rates among strains, we compared the time points when *m*(*t*) and *r*(*t*) reach their maximums, denoted as T_mmax_ and T_rmax_, respectively. T_mmax_ and T_rmax_ values for all 11 natural isolates are strongly correlated (R^2^ = 0.60, p-value = 0.005, and Table 2). Overall, T_rmax_ is 1.1±0.2 fold of T_mmax_. We conclude that LOH in chronologically aged mother cells not only increases in fraction, shown by *b*(*t*), but its rate of occurrence also increases up to the point of T_rmax_, shown by *r*(*t*). Hence, cells shift to a high level of genomic instability during chronological aging.

### Change of LOH in daughters cells

Half-black colonies indicate the occurrence of LOH events after the first cell division on plates (Figure 1). We quantified the fractions of half-black colonies during chronological aging for the 11 natural isolates. Because half-blacks can only be generated by parental wild-type cells, we calculated the fraction of half-blacks relative to parental cells, denoted as *b_(1/2)_* (detailed in Methods). The results for strain M5 are shown in Figure 2C. The fraction of half-blacks over time mirrors the fraction of full-blacks, and shows a dramatic increase late in the aging time course. Note that in Figure 2C the maximal fraction of half-blacks (∼0.05) is about 8% the maximal fraction of full-blacks (∼0.4) in Figure 2A. Whereas the fraction of full-blacks among aging populations of the 11 natural isolates generally plateaus, the fraction of half-blacks can peaks substantially (Figure S1). The peak is so strong in some strains that approximation by logistical modeling becomes difficult. For simplicity, we recorded the time point when *b*_1/2_(*t*) reaches its maximum, denoted as T_dmax_. A strong demographic-wide correlation was observed among the 11 natural isolates between the loss of viability, T_c_, and the peak production of half-black LOH events, T_dmax_ (R^2^ = 0.71, p-value = 0.001, and Table 2).

To further examine the changes of half-blacks and full-blacks, we compared the changes in their frequencies over time (Figure 2D). The total number of full-black cells drops as the entire population dies off during aging. However, there is a peak of half-blacks, indicating a sudden burst in the number of stationary mother cells that give rise to half-black colonies. The timing of the burst is around T_dmax_, the time when the fraction of half-blacks peaks. Although half-blacks can be due to LOH in either mother or daughter cells, the burst in half-blacks, but not in the full-blacks, argues that the age-dependent increase of half-blacks is in large portion due to the sharp increase of LOH in daughter cells.

### Mitotic asymmetry breaks down during chronological aging

The asymmetric partitioning of damaged macromolecules to mother cells during cell division is thought to be at least partially responsible for the phenomenon of daughter cells being born young while their mother cells age [7], [8], [12]. This mitotic asymmetry breaks down during replicative aging and leads to elevated LOH in daughter cells [10], [11]. To investigate the effect of chronological aging on mitotic asymmetry, we calculated the ratio
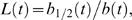
(6)where *b_1/2_*(t) is the chance parental wild-type cells will become half-blacks, and *b*(*t*) is the chance they will become full-blacks. *L*(*t*) is only proportional to mitotic asymmetry, because *b_1/2_*(*t*) includes LOH events occurred in both mother and daughter cells after the first mitosis and before the second mitosis (Figure 1). The peak in the frequency of half-blacks but not full-blacks indicates that most of the half-blacks are due to LOH events in daughter cells late in aging (Figure 2D). Hence, the fluctuation of *L*(*t*) reflects change of mitotic asymmetry during aging. As shown for strain M5 in Figure 2E, *L*(*t*) shows a strong peak during the die-off phase of chronological aging. *L*(*t*) peaks for all 11 natural isolates (Table S1, and Figure S2). The peak of *L*(t) is due both to the peak of *b_1/2_*(*t*) as described above and a slower increase in the fraction of full-blacks, *b*(*t*).

Peaks of *L*(*t*) often occur after the mean life span T_c_. We denote the time that *L*(t) peaks as T_Lmax_. On average, T_Lmax_ is 1.3±0.6 fold of T_c_. Pairwise t-test shows that the difference between T_Lmax_ and T_c_ is significant at a p-value of 0.009.

Interestingly, the initial *L*(t = 0) is negatively correlated with CLS measured previously on plates without Pb^2+^ (R^2^ = 0.53, p = 0.01, Table 2), which indicates that strains with more half-blacks at time zero, and hence weaker mitotic asymmetry, tend to have shorter CLS. This correlation further demonstrates that *L*(t) is a useful proxy for mitotic asymmetry.

### Ability of natural isolates to tolerate genomic instability during chronological aging co-varies with replicative life spans

Above we showed a striking increase in genomic instability as measured by LOH during chronological aging in both mother cells, indicated by the full-black colonies, and the daughter cells, indicated by the half-black colonies. We found that increase of LOH during aging, indicated by T_g_, are generally slower than decrease of viability, indicated by T_c_. This gap suggests that aging cells is able to hold off damaging effects of genotoxic stress during aging. Hence, we sought to determine whether the ability of yeast strains to maintain genome integrity is correlated with natural variation in the ARLS of the same 11 natural isolates.

**Figure 3:**
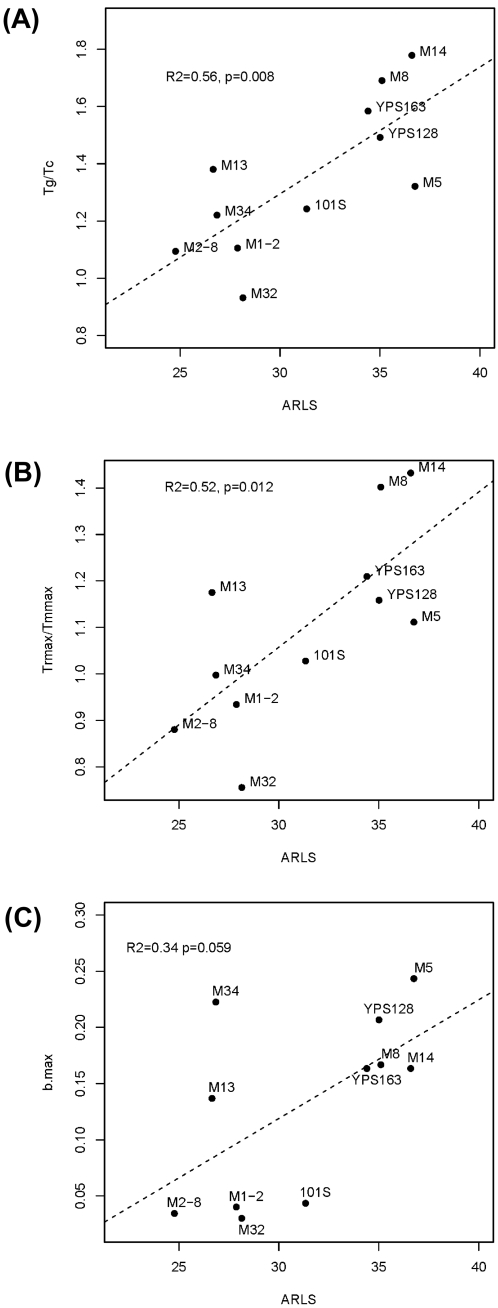
Ability to counter genomic instability is associated with ARLS at the demographic level. Three different proxies for tolerance to genomic instability are presented: T_g_/T_c_ in (A), T_rmax_/T_mmax_ in (B) and b_max_ in (C). Values for each strain are the averages of at least 3 experiments.

We first used the ratio of T_g_/T_c_ to quantify the ability of each natural isolate to counter genotoxic stress associated with aging. Linear regression shows that ARLS is positively correlated with this measure with p-value = 0.008 and R^2^ = 0.56 (Figure 3A). Hence, the lag between T_c_ and T_g_ accounts for more than 50% of the observed natural variation in replicative life span.

We then used a second measure, T_rmax_/T_mmax_, the ratio between the time points that the mortality rate and the genome instability rate reach their maximums, respectively. This second measure based on rates is conceptually related to the first measure based on cumulative changes. As shown in Figure 3B, ARLS is also positively correlated with T_rmax_/T_mmax_ (p-value = 0.01, R^2^ = 0.52). Hence, the second measure also accounts for more than 50% of the natural variation in replicative life span.

Finally, we used a third measure, b_max_, the maximal percentage of full-black colonies which indicates the maximal genomic instability a strain can tolerate. Regression between ARLS∼b_max_ shows a positive correlation with p-value = 0.059 and R^2^ = 0.32 (Figure 3C). To further verify this correlation, we partitioned strains into two groups with long ARLS (>33) and short ARLS (≤33). Strains with long ARLS indeed have high levels of b_max_ (t-test, p-value = 0.02). Near the end of CLS assays, it becomes increasingly difficult to obtain large number of colonies, which results in greater fluctuations than initial stages. Because b_max_ is measured at late stages of aging, it is more susceptible to experimental fluctuations than are the first two measures. Nevertheless, b_max_ still accounts for more than 30% of the natural variation in RLS.

Taken together, we conclude that ability of cells to maintain genome integrity during chronological aging accounts for 30∼50% of the natural variation in RLS. Regressions were also performed for several other factors (See Table 2 for summary), including CLS measured on plates without Pb^2+^. CLS was not significantly correlated with T_g_/T_c_ , T_rmax_/T_mmax_ or b_max_.

### *RAD52* is not required for the age-dependent increase of genomic instability

Break-induced DNA replication (BIR) is the primary pathway for elevated LOH during replicative aging [10], [11]. Rad52 is a strand annealing protein that is required in homologous recombination repair of double-strand break (DSB) in DNA, including BIR [23]. Therefore, we sought to determine if Rad52 plays an important role in the control of LOH during CLS.

**Figure 4:**
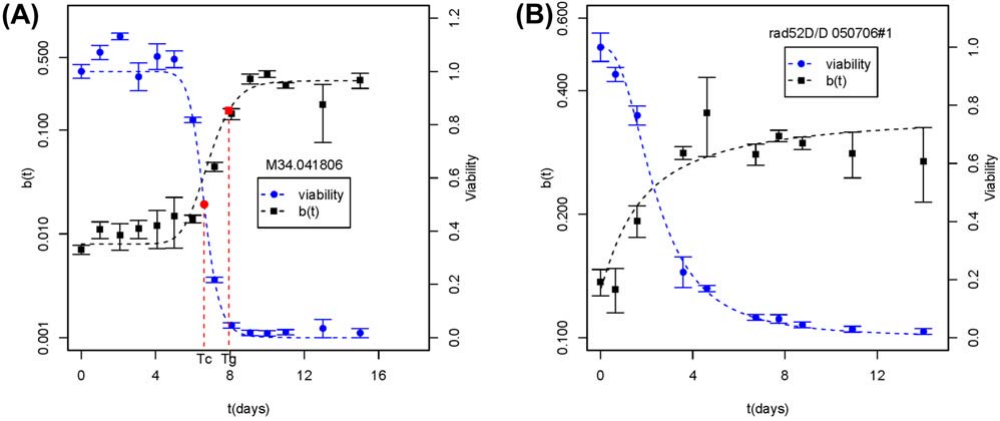
Effect of *rad52^−/−^* on LOH during chronological aging. Change of full-black LOH during chronological aging in the parental strain M34 (A) and in the *rad52* ^−/−^ derivative (B). *RAD52* is not essential for the age-dependent increase of LOH. Viability *s*(*t*) and fraction of black colonies *b*(*t*) are drawn in different scales.

As shown in Figure 4, the increase of LOH over time is clearly observable in the absence of *RAD52*. The basal level of LOH is increased dramatically in *rad52^−/−^* mutant cells, presumably due to a higher level of DSBs. The increase of LOH is not as sigmoid as that in the wild-types, but this may be due to the rapid loss of viability of the mutant cells. Overall, although Rad52 is required to repair damage that otherwise would lead to LOH events, *RAD52* is not essential for the age-dependent rise of LOH events during chronological aging.

## Discussion

In this study, we provide the first quantitative study on genomic instability and natural life span variation and we report a striking increase in genomic instability during chronological aging in the form of LOH in both mother and daughter cells. By quantifying age-dependent changes of LOH and CLS in a collection of natural isolates, we found that the increase of genomic instability is in general slower than the loss of viability. The capacity of yeast stains to counter genomic instability, shown by T_g_/T_c_, T_rmax_/T_mmax_ and b_max_, accounts for 30∼50% variation in ARLS for these natural isolates, which shows that a capacity to better maintain genomic integrity leads to longer RLS.

Why does the ability to maintain genome integrity co-vary with replicative age? It is possible that genomic instability measured at the *MET15* locus is influenced by the nearby rDNA locus. Accumulation of the extrachromosomal rDNA circles is a demonstrated cause of yeast replicative aging [24]. Therefore, strains with better ability to maintain integrity of the rDNA locus tend to have longer RLS. The rDNA locus is also involved in DNA replication stress, which has been implicated in both replicative and chronological aging [25], [26]. This speculated role of the rDNA locus certainly does not preclude other possibilities. In fact, an un-biased approach such as search for quantitative trait loci (QTL) may be the most cost-effective way to address the underlying genetic mechanisms for natural variations [27]–[29].

What causes LOH to lag after the loss of viability during aging? Recall that ARLS is the average number of cell divisions that cells can accomplish. Correlation between ARLS∼T_g_/T_c_ suggests that the lag may be due to a cell-cycle related mechanism. It has been shown that cells are in heterogeneous cell-cycle states when OD plateaus [30], [31]. This heterogeneity and the potential interdependency of cell subpopulations may be part of the orchestrated response of cells to starvation. It is plausible that the lag between T_c_ and T_g_ is caused by cell subpopulations in different cell-cycle states, and the variation of this lag in natural isolates of yeast may be attributed to the characteristic distribution and interdependency of cell subpopulations in each yeast isolate. Another possibility may be related to the slowing down of cell-cycle during aging. The prolonged G2/M phase in aged cells has been speculated to be involved in the change of DSB repair methods during aging in both yeast and fruit fly [10], [32]. We argue that further understanding of this question demands QTL study on natural variation, which can shed lights not only on the lag but also on the cause of age-induced LOH.

There are some caveats in our current study, especially the effect of Pb^2+^, the possibility of sporulation, and the influence of regrowth. The nature of our LOH assay makes it hard to separate the effect of aging on genomic integrity from the possible effect of Pb^2+^ on genomic integrity. However, previous studies in both yeast and fruit flies show that experimental schemes with stressful conditions, including Pb^2+^, high temperature and paraquat, led to useful information on aging [10], [33]–[35].

Due to the diploid nature of the strains used, there is a possibility of sporulation during the aging process. However, the potential sporulation cannot be a major factor on the section patterns of colonies due to following reasoning. (1) Spores are supposed to live for a very long time. Therefore their frequency over time should increase due to accumulation, not decline as the full-blacks or spike as the half-blacks in Figure 2D. (2) Significant breakdown of the asci is required for sporulation to account for the dominant full-black colonies. Due to the sticky morphology of natural yeast isolates, we used sonication pulses to disperse cell clumps, which could break up the asci. To address this problem, we performed the same experiments using the laboratory strain BY4743, which does not need sonication due to its smooth morphology. Similar frequency spectrums of full- and half-blacks during aging were observed and the full-blacks remain as the dominant section pattern except in the peak region of the half-blacks (Figure S3). (3) It is not straightforward how random breakup of asci and random association among spores can result in different frequency spectrums of full- and half-blacks and the spike of L(t).

In about one out of three CLS assays (12 out of 35 experiments), we observed regrowth after viability drops to 0.01∼0.1%, similar to other reports [3], [36], [37]. When regrowth happens, most of the newly grown colonies are white ones, suggesting that cells with LOH are less fit than those without LOH. For simplicity, we have excluded data points with regrowth, because we are interested in the effect of aging on LOH. The plateau of full-black colonies is not caused by regrowth, because the fraction of full-black colonies actually drops sharply when regrowth occurs. Furthermore, we observe the sigmoid shape of full-blacks in fraction in all experiments.

Yeast aging is relevant to aging in higher organisms, as supported by the conserved role of calorie restriction in yeast, worm, fruit fly, mouse, and human [4], [38]–[42]. At the biodemographic level, a negative correlation between the log-transformed initial mortality rate and the Gompertz coefficient can be observed in both mammals and yeast [20], [43]. Hence, our findings may provide insights into life span plasticity in other organisms.

## Materials and Methods

### Yeast strains

Natural isolates were detailed previously [20]. Derivatives of natural isolates of *Saccharomyces cerevisiae* used in this study are summarized in Table S2. One copy of the *MET15* wild type allele was knocked by long-arm PCR strategy with kanamycin-resistence (*kan^r^*) as a selection marker.

We used a diploid homozygous derivative of M34 to generate *MET*15^+/−^ and *rad52* ^Δ/Δ^ strain. First, we knocked out one copy of *HO* gene in the parental M34 strain with a *kan* ^r^ marker flanked by lox-P sites. After sporulation, haploid progenies were identified. Spontaneous *ura3* ^−^ mutants were obtained based on resistance to FOA. These *ho* ^Δ^, *ura*3^−^ M34 haploid strains were then transformed with a plasmid carrying *CRE*, and the *kan* ^r^ marker was removed. Long-arm PCR and NAT-resistance marker were used to knock out *RAD52* in the haploid strains for both mating types. *MET15* in one of haploid strains was subsequently knocked out using the *kan* ^r^-marker. Finally, haploids were mated, potential diploids cells were identified based on colony size and morphology (Diploid colonies are larger and more smooth than haploid ones). Potential diploid colonies were spread on to Pb^2+^-containing plates. Those with sectioning patterns are identified as diploids cells and should have the genotype *ho* ^Δ/Δ^, *MET*15^+/Δ^, *rad52* ^Δ/Δ^. These candidates are finally verified by PCR followed by agarose gel electrophoresis.

### LOH in yeast chronological aging

Yeast strains were grown in liquid YPD with 2% glucose to the point that its growth rate has reached plateau based on OD measures [13], [20]. Yeast cells were then washed three times, spun down, and transferred to water. Cells were adjusted to 2∼6×10^8^ cells/ml and kept in rotation at 50 rotations per minute at 30°C. Aliquots of yeast cells were taken at different time intervals. Aliquots were chilled on ice for 15 minutes, sonicated at low power setting for 30 seconds, and then immediately chilled on ice. The aliquots were then diluted to 3 different concentrations and were spread on Pb^2+^-containings plates [21]. The level of Pb(NO_3_)_2_ in the plates was 0.7% [10]. We aimed to obtain 1000∼3000 colonies on each 150 mm-diametered plate. After 2 days of incubation at 30°C, plates were moved to room temperature for 2–3 days in order for colors to darken. Colonies were scored as white, full-black, half-black, quarter-black, or 3/4-black. Digital images of plates were taken for records.

### Estimation of LOH and aging

Induction of the LOH model is presented in next section. For LOH in half-black colonies, its raw percentage has to be normalized, because half-blacks can only come from heterozygous mother cells. We calculated *b*_(1/2)_ as
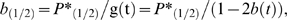
where P*_(1/2)_ is the unadjusted percentage of half-blacks calculated based on total colony counts. The denominator represents the percentage of wild-type cells at a given time. This adjustment becomes increasingly important in late stage of aging when *b*(*t*) has increased significantly.

The mortality rate is calculated as:
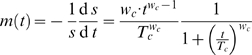



The rate of genomic instability is calculated as:
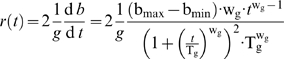



Numerical and statistical analysis was performed in the R language and environment for statistical computing (http://www.r-project.org/). Sample scripts are available from HQ upon request.

### Modeling the genomic instability during chronological aging

To derive the model for *b*(*t*), we start with a general form for genomic integrity *g*(*t*):
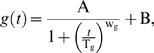
(7)where T_g_ is the time when the decrease of genomic integrity has reached 50% of its total extent of changes, w_g_ is a weight parameter, and A and B are two constants yet to be determined. In addition, we know the following conditions hold:
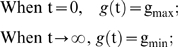
where g_max_ and g_min_ are the maximum and minimum of *g*(t). Parameters A and B can be solved from the above two equations. By substitution of A and B in the general form, we obtain the following formulation for genomic integrity:
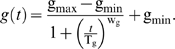
(8)We can verify that *g*(*t* = T_g_) = g_max_/2+g_min_/2, which is the midpoint between g_max_ and g_min_.

A similar form can then deduced for *b*(*t*), the percentage of full-black colonies over time. If we assume equal chances of LOH for *MET*15^+/+^ and *met*15^−/−^, we have:
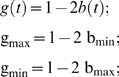
Plug the above three equations into Eq 8, we have the model for *b*(*t*):
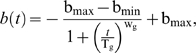
which is Eq 3. We can also verify that *b*(*t* = 0) = b_min_, *b*(*t*→∞) = b_max_, and *b*(*t* = T_g_) = b_max_/2+b_min_/2.

## Supporting Information

Figure S1 An example that b_1/2_(t) can peak significantly during aging. An experiment is presented for strain M14. Error bars indicate standard deviation calculated from 3 plates.
(0.76 MB TIF)

Figure S2 The peaks of L(t) during chronological aging are presented. Five examples are presented. The peaks are more conspicuous in some experiments than in others.
(3.63 MB TIF)

Figure S3 Frequency spectrums of full- and half-blacks in BY4743 during chronological aging. The diploid laboratory strain BY4743 has a smooth morphology and does not need sonication to disperse cells. This experiment show that in the absence of sonication, the frequency of half-blacks still peak around the time when viability drops, the full-blacks still out-number the half-blacks, and both full-blacks and half-blacks frequency decrease as viability further drops. These observations are similar to those in natural isolates and are inconsistent with the argument that sporulation is the major cause of the sectioned colonies. Standard deviations are presented in the plots.
(0.47 MB TIF)

Table S1 Summary of key estimations.
(0.06 MB DOC)

Table S2 Yeast strains used in this study.
(0.06 MB DOC)
