# Supplementary figures and images for: Genomic Instability Is Associated with Natural Life Span Variation in Saccharomyces cerevisiae
Source: PLoS One. 2008 Jul 16;3(7):e2670. doi: 10.1371/journal.pone.0002670 (PMC2441830; doi:10.1371/journal.pone.0002670)

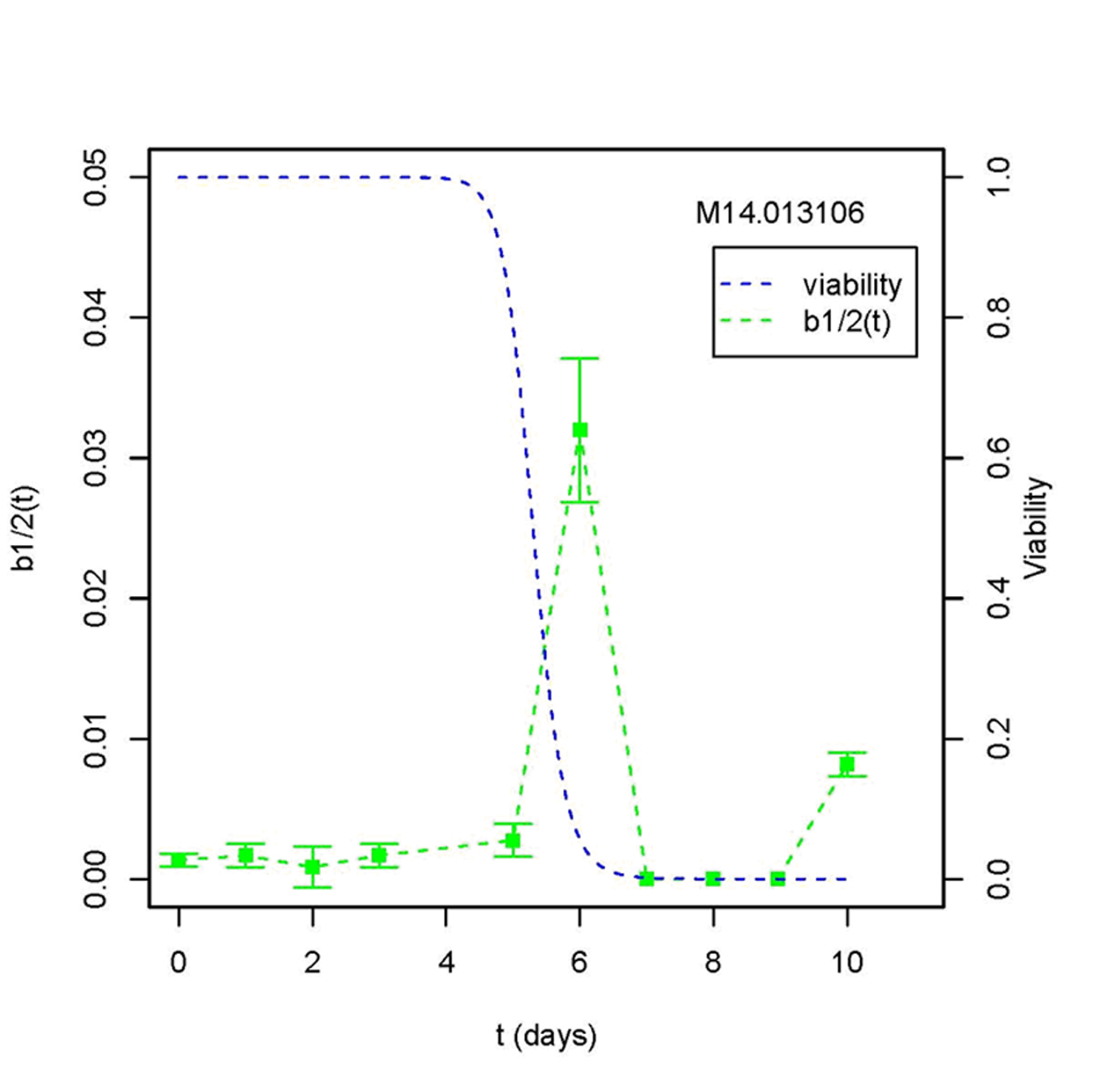

Supplement: Figure S1 — An example that b1/2(t) can peak significantly during aging. An experiment is presented for strain M14. Error bars indicate standard deviation calculated from 3 plates. (0.76 MB TIF) [file pone.0002670.s001.tif]

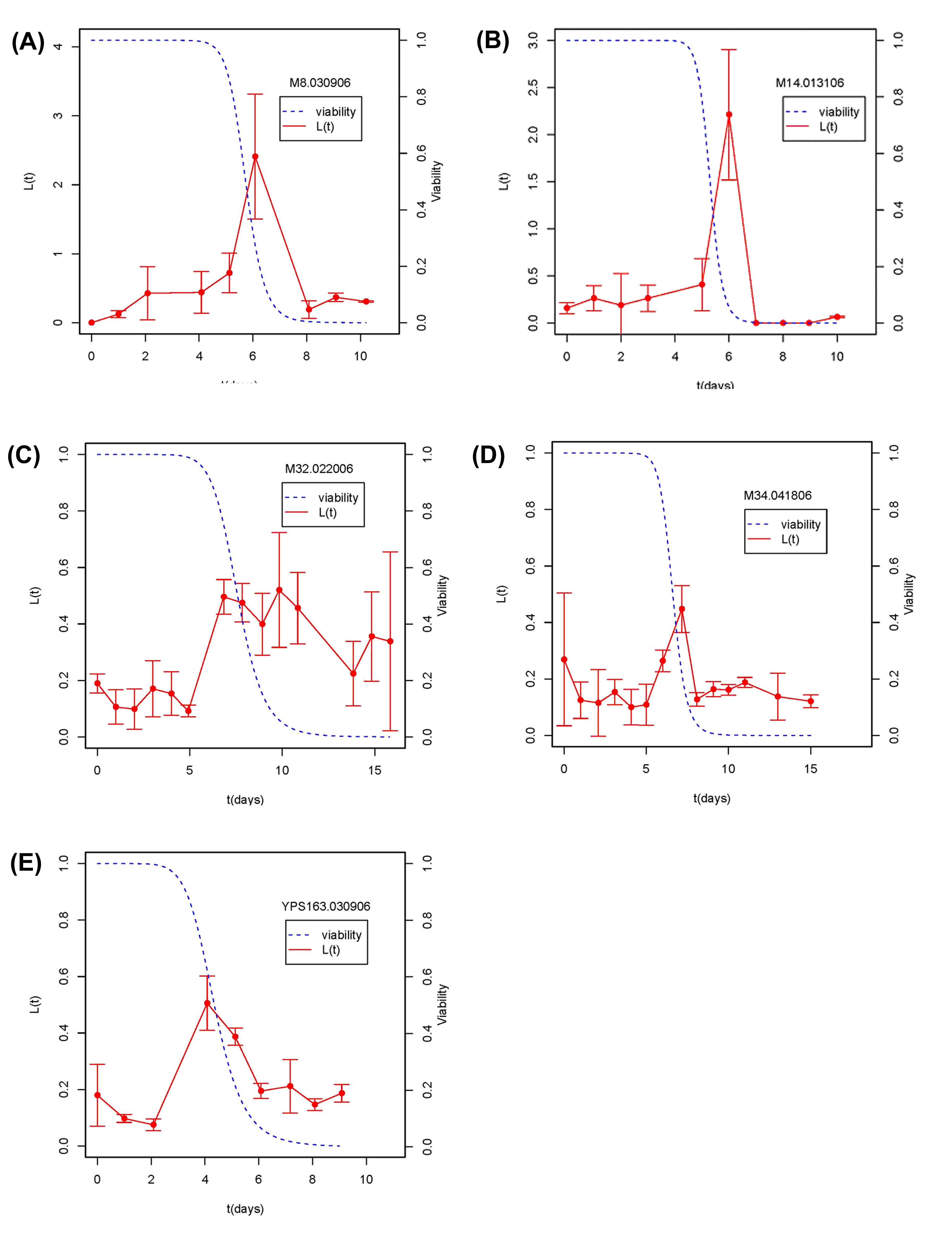

Supplement: Figure S2 — The peaks of L(t) during chronological aging are presented. Five examples are presented. The peaks are more conspicuous in some experiments than in others. (3.63 MB TIF) [file pone.0002670.s002.tif]

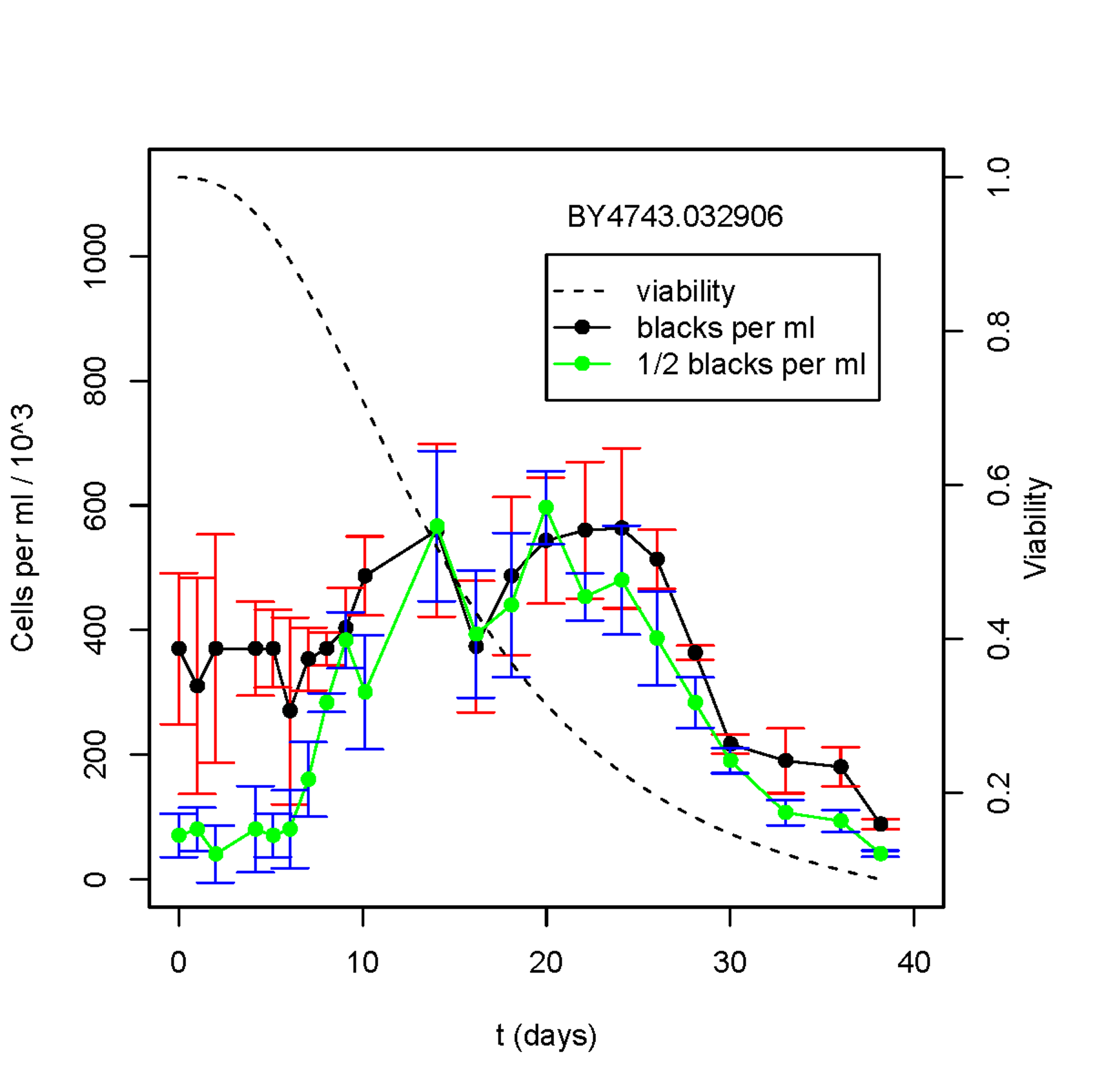

Supplement: Figure S3 — Frequency spectrums of full- and half-blacks in BY4743 during chronological aging. The diploid laboratory strain BY4743 has a smooth morphology and does not need sonication to disperse cells. This experiment show that in the absence of sonication, the frequency of half-blacks still peak around the time when viability drops, the full-blacks still out-number the half-blacks, and both full-blacks and half-blacks frequency decrease as viability further drops. These observations are similar to those in natural isolates and are inconsistent with the argument that sporulation is the major cause of the sectioned colonies. Standard deviations are presented in the plots. (0.47 MB TIF) [file pone.0002670.s003.tif]
