# Supplementary material for: Genomic Instability Is Associated with Natural Life Span Variation in Saccharomyces cerevisiae
Source: PLoS One. 2008 Jul 16;3(7):e2670. doi: 10.1371/journal.pone.0002670 (PMC2441830; doi:10.1371/journal.pone.0002670)
Supplement: Table S1 — Summary of key estimations. (0.06 MB DOC) [file pone.0002670.s004.doc]

Table S1. Summary of key estimations.

| Strain | N | T_c_ | T_g_ | T_mmax_ | T_rmax_ | T_dmax_ | T_Lmax_ | b_max_ | L_0_ | L_max_ |
| --- | --- | --- | --- | --- | --- | --- | --- | --- | --- | --- |
| 101S | 3 | 4.6±0.4 | 5.7±0.9 | 5.5±0.4 | 5.6±0.9 | 4.8±0.4 | 6.7±1.1 | 0.04±0.02 | 0.16±0.20 | 2.5±0.5 |
| M1-2 | 3 | 6.3±0.4 | 6.9±0.9 | 7.3±0.5 | 6.8±0.9 | 6.9±0.9 | 7.7±1.5 | 0.04±0.03 | 0.06±0.15 | 0.7±0.3 |
| M13 | 3 | 8.0±0.3 | 11.1±1.0 | 9.4±0.4 | 11.1±1.0 | 9.3±1.5 | 9.7±2.7 | 0.14±0.02 | 0.09±0.20 | 0.7±0.5 |
| M14 | 3 | 4.0±1.2 | 7.0±0.5 | 4.9±1.0 | 7.0±0.5 | 5.84±1.9 | 9.5±3.0 | 0.16±0.05 | 0.11±0.11 | 1.2±0.9 |
| M2-8 | 4 | 4.1±0.7 | 4.5±0.4 | 5.1±0.8 | 4.5±0.5 | 5.9±1.7 | 7.0±1.4 | 0.03±0.02 | 0.15±0.21 | 3.3±2.9 |
| M32 | 3 | 8.0±0.7 | 7.4±1.8 | 9.7±1.6 | 7.4±1.8 | 8.2±0.7 | 7.6±1.5 | 0.03±0.01 | 0.11±0.09 | 0.6±0.1 |
| M34 | 4 | 6.7±1.5 | 8.2±1.3 | 8.3±2.0 | 8.3±1.3 | 8.4±0.6 | 7.6±1.9 | 0.22±0.12 | 0.17±0.19 | 0.8±0.4 |
| M5 | 3 | 5.9±0.4 | 7.8±1.5 | 7.1±0.3 | 7.8±1.7 | 6.5±0.6 | 6.9±0.9 | 0.24±0.15 | 0.17±0.36 | 1.8±1.3 |
| M8 | 3 | 6.8±2.4 | 11.5±4.4 | 8.2±3.0 | 11.5±4.4 | 6.0±2.0 | 6.9±1.9 | 0.17±0.15 | 0.06±0.12 | 1.2±1.0 |
| YPS128 | 3 | 8.6±3.0 | 12.9±4.6 | 10.8±3.2 | 12.5±4.0 | 11.4±5.7 | 11.8±6.2 | 0.21±0.17 | 0.18±0.13 | 1.7±1.3 |
| YPS163 | 3 | 5.2±0.8 | 8.3±2.7 | 6.9±1.2 | 8.3±2.7 | 7.1±3.3 | 5.2±2.3 | 0.16±0.06 | 0.25±0.10 | 1.3±1.0 |

# N is the number of experiments performed. A different transformant obtained from strain construction was also used (except for M5). All strains contain the *MET*15^+/-^ marker. Standard deviations are provided.
