# Supplementary material for: Genomic Instability Is Associated with Natural Life Span Variation in Saccharomyces cerevisiae
Source: PLoS One. 2008 Jul 16;3(7):e2670. doi: 10.1371/journal.pone.0002670 (PMC2441830; doi:10.1371/journal.pone.0002670)
Supplement: Table S2 — Yeast strains used in this study. (0.06 MB DOC) [file pone.0002670.s005.doc]

Table S2. Yeast strains used in this study

| **Strain** | **Description** | Source |
| --- | --- | --- |
| 101S *MET*15^+/-^ | Parental strain is 101S. | This study |
| M1-2 *MET*15^+/-^ | Parental strain is M1-2. | This study |
| M13 *MET*15^+/-^ | Parental strain is M13. | This study |
| M14 *MET*15^+/-^ | Parental strain is M14. | This study |
| M2-8 *MET*15+/- | Parental strain is M2-8. | This study |
| M32 *MET*15^+/-^ | Parental strain is M32. | This study |
| M34 *MET*15^+/-^ | Parental strain is M34. | This study |
| M5 *MET*15^+/-^ | Parental strain is M5. | This study |
| M8 *MET*15^+/-^- | Parental strain is M8. | This study |
| YPS128 *MET*15^+/-^ | Parental strain is YPS128. | This study |
| YPS163 *MET*15^+/-^ | Parental strain is YPS163. | This study |
| M34 *MET*15^+/-^ *rad52* ^Δ /Δ^ | Parental strain is M34. | This study |
| BY4743 *MATa/α*, *his3*^Δ /Δ^, *lecu2*^Δ /Δ^, *ura3*^Δ /Δ^, *MET*15^+/-^ , *LYS2*^+/-^ | Diploid laboratory strain | Euroscarf Y20000, http://web.uni-frankfurt.de/fb15/mikro/euroscarf/ |
